# Accurate 3D maize ear phenotyping using voxel grids derived from RGB machine vision

**DOI:** 10.1016/j.plaphe.2026.100274

**Published:** 2026-08-26

**Authors:** Leon H. Oehme, Tobias Schrag, Peng Qi, Bastian Stürmer-Stephan, Khandoker T. Ahammad, Daniel Wanke, Tobias Würschum, Xiongkui He, Joachim Müller

**Affiliations:** a Tropics and Subtropics Group, Institute of Agricultural Engineering, University of Hohenheim, Stuttgart, 70599, Germany; b Institute of Plant Breeding, Seed Science and Population Genetics, University of Hohenheim, Stuttgart, 70599, Germany; c College of Science, China Agricultural University, Beijing, 100193, China; d Institute of Agricultural Engineering, University of Hohenheim, Stuttgart, 70599, Germany; e Institute of Crop Science, Fertilisation and Soil Matter Dynamics, University of Hohenheim, Stuttgart, 70593, Germany

**Keywords:** Maize, Phenotyping, Three-dimensional, Voxelization, Segmentation, Deep learning, Phosphorus

## Abstract

Maize ear phenotyping is receiving increasing attention, driven by the demand of plant scientists for large-scale trait data and enabled by the rapid development of AI-based machine vision. While manual measurement of ear traits is labour-intensive and often destructive, existing digital solutions typically rely on a limited number of 2D images without capturing the entire 3D structure of an ear. Therefore, a novel 3D ear scanning pipeline was developed that computes voxel grids based on RGB images automatically captured from 100 different viewing angles. For kernel segmentation, open-source deep learning models for object detection and segmentation were evaluated and combined, achieving a dice score of 0.94 on 2D ear images. A novel strategy for mapping 2D segmentation masks to 3D-oriented voxels resulted in high coefficients of determination (*R*^*2*^) for predicting kernel number (*R*^*2*^ = 0.98), kernel row number (*R*^*2*^ = 0.77) and maximum kernel row length (*R*^*2*^ = 0.87). Similarly, ear width (*R*^*2*^ = 0.82), ear length (*R*^*2*^ = 0.96) and ear volume (*R*^*2*^ = 0.97) were accurately predicted when compared to manual reference measurements. The applicability of the pipeline was demonstrated on ears grown under two levels of phosphorus supply, which revealed significant effects on ear width, ear length, ear volume and kernel size. Data acquisition required 30 s per ear, while computation of all traits took an average of 47 s. This study establishes an accurate and fast 3D phenotyping pipeline while bridging high-throughput ear phenotyping with plant nutrition research, demonstrating its potential to link structural traits with nutrient-driven responses in crops.

## 1 Introduction

Maize (*Zea mays* L.) is one of the most widely grown crops worldwide, playing a central role in human nutrition, animal feed production, and industrial applications. Research on maize often relies on large amounts of phenotypic data, which motivated an increasing number of high-throughput phenotyping (HTP) applications in recent years [1]. Especially plant breeding benefits from HTP by allowing to link genomic data to phenotypic data on a relevant scale. Manual phenotyping has traditionally been a major bottleneck in plant breeding, which can be overcome by HTP solutions [2]. Other disciplines of plant science that greatly benefit from HTP in maize are plant physiology [3], plant pathology [4] and plant nutrition [5]. Numerous HTP studies have focused on maize plant traits under both greenhouse conditions (e.g. Daviet et al. [3]) and field conditions (e.g. Oehme et al. [6]). Furthermore, increasing attention is being directed toward HTP of the maize ear, which represents the most nutritionally valuable organ of the plant. In contrast to maize plant architecture, which is typically studied on fresh plants, maize ear architecture does barely change after drying, which greatly facilitates automatic HTP approaches.

Although the feasibility of maize ear phenotyping has been demonstrated in multiple studies, the approaches differ greatly in the type of input image data and the traits that were studied. Often, only a single or a limited number of 2D RGB images were used for analysis, without exploiting the spatial relationship between the camera and the maize ear. Miller et al. [7] used 2D RGB images to predict kernel spacing on the ear, while kernel dimensions and kernel count were estimated only after the kernels had been removed. Additional ear traits, such as kernels per row, kernel row number (KRN) and kernel number (KN) were identified per single view RGB image in Shi et al. [8]. Makanza et al. [9] measured ear size, ear number, kernel size (KS) and KN on single RGB images captured by a portable phenotyping setup. The work of Kienbaum et al. [10] focused on ear morphological traits as retrieved from RGB images. In the recent work of Ma et al. [11], HTP of maize ear phenotyping was applied for sorting of maize ears, which was enabled by efficient processing of 2D image data. Zaremehrjerdi et al. [12] presented MaizeEar-SAM, which utilizes the segment anything model (SAM) [13] for kernel segmentation and subsequently incorporates graph theory to compute kernels per row, again based on 2D image data. Single views of 2D image data were also the base for Ma et al. [14] to predict ear volume (EV). Oury et al. [15], however, obtained multiple views per ear by capturing ears on rotating rollers. Although this allowed accurate prediction of traits such as ear length (EL), ear width (EW) and KN, their method lacked orienting the 2D views in the ear's 3D coordinate system.

In contrast, Warman et al. [16] captured single pixel rows depicting the centre of a rotating ear via a continuous video stream, ensuring that all sides of the maize ear are present in the analysis. Thereby all kernels of an ear could be counted with an R^2^ of up to 0.999 when compared to manual counting. Kirchgessner et al. [17] proposed the FruitPhenoBox equipped with five cameras, that capture single apples. Despite the limited number of views, the stationary setup ensures that the views are equally distributed around the apple and can be oriented in relation to the apple's 3D coordinate system. 3D oriented phenotypic information of maize kernels is directly provided by the computed tomography data processed in Zhao et al. [18]. To detect maize ear rot, Yang et al. [4] computed 3D point clouds from the multi-view stereo algorithm. While having generated accurate 3D representations of maize ears, they do not report results on ear or kernel morphological traits. Although no results on EV were reported, the recent work of Sun et al. [19] established the 3D spatial orientation of individual kernels and assigned them to kernel rows based on this geometry.

Despite significant advancements in single-view-based ear phenotyping, comprehensive and precise high-throughput 3D phenotyping of maize ears, which concurrently assesses the relevant 3D traits KRN, EV, and KN across the whole ear surface, remains limited. In this work, a novel voxel-based approach is proposed to accurately and efficiently determine maize ear traits oriented in 3D space. The main objectives in this study are:

i. Training and establishing an instance segmentation approach to accurately segment maize kernels on 2D images;
ii. Evaluating prediction quality of multiple ear and kernel morphological traits based on manual reference measurements;
iii. Demonstrating applicability of the established ear scanning pipeline on ears from a phosphorus (P) fertilization trial.

## 2 Materials and methods

### 2.1 Plant material

A total of 105 morphologically diverse ears were sampled from a larger field trial described in Haller et al. [20]. The ears resulted from 78 testcrosses of Flint lines (elite or landrace double-haploids) and 9 commercial hybrids. Multiple common ear traits were manually measured on these ears to serve as reference dataset. Another 90 ears were sampled from the 2023 field trial described in Herrmann et al. [21]. Here, the commercial variety LG30258 was grown on P deprived soil supplied with different P treatments in a three-factorial Latin-square split-plot design. A total of 45 ears originated from plots supplied with no P fertilizer, serving as control, while another 45 ears were sampled from plots supplied with diammonium phosphate fertilizer (DAP) (18% N, 20.1% P) as broadcast application. Despite preparatory cleaning, one ear from the unfertilized control group had to be excluded from the experiment due to heavy abundance of pollen tubes which led to occlusion of the ear. That left 44 ears of the unfertilized control and 45 ears of the P treatment to demonstrate applicability of the ear scanning pipeline.

### 2.2 Manual trait measurements

Multiple ear traits were manually measured for all 105 ears of the reference dataset. EL and EW were measured as the widest distance between ear tip and ear base and the widest ear diameter, respectively. Only kernels that were expected to be able to germinate were counted for KN. Already counted kernels were painted with a marker to avoid counting them more than once. KRN was determined by counting the number of longitudinal kernel rows on the ear. To be included, the kernel rows could stretch from ear base to ear tip or could be incomplete due to missing kernels. Moreover, kernels of the longest undisrupted kernel row between ear base and ear tip were counted to determine the kernel row length (KRL).

EV was measured based on water displacement inside a cylinder. The cylinder was filled with water up to maximum capacity, to then submerge the ear with a pair of tweezers. Submerging the ear leads to water overflowing the cylinder leaving behind a smaller amount of water that then can be weighed and subtracted from the cylinder weight at maximum capacity:

(1) $$\begin{array}{c} EV=\frac{m_{0}-m_{1}}{\rho }\text{,} \end{array}$$

where *m*_*0*_ is the mass of the cylinder filled with water to maximum capacity and *m*_*1*_ is the mass of the cylinder after displacement of water caused by submerging the ear. The mass difference is divided by *ρ* which is the density of water at 20°C to translate mass to volume.

### 2.3 Ear scanning setup

**Fig. 1:**
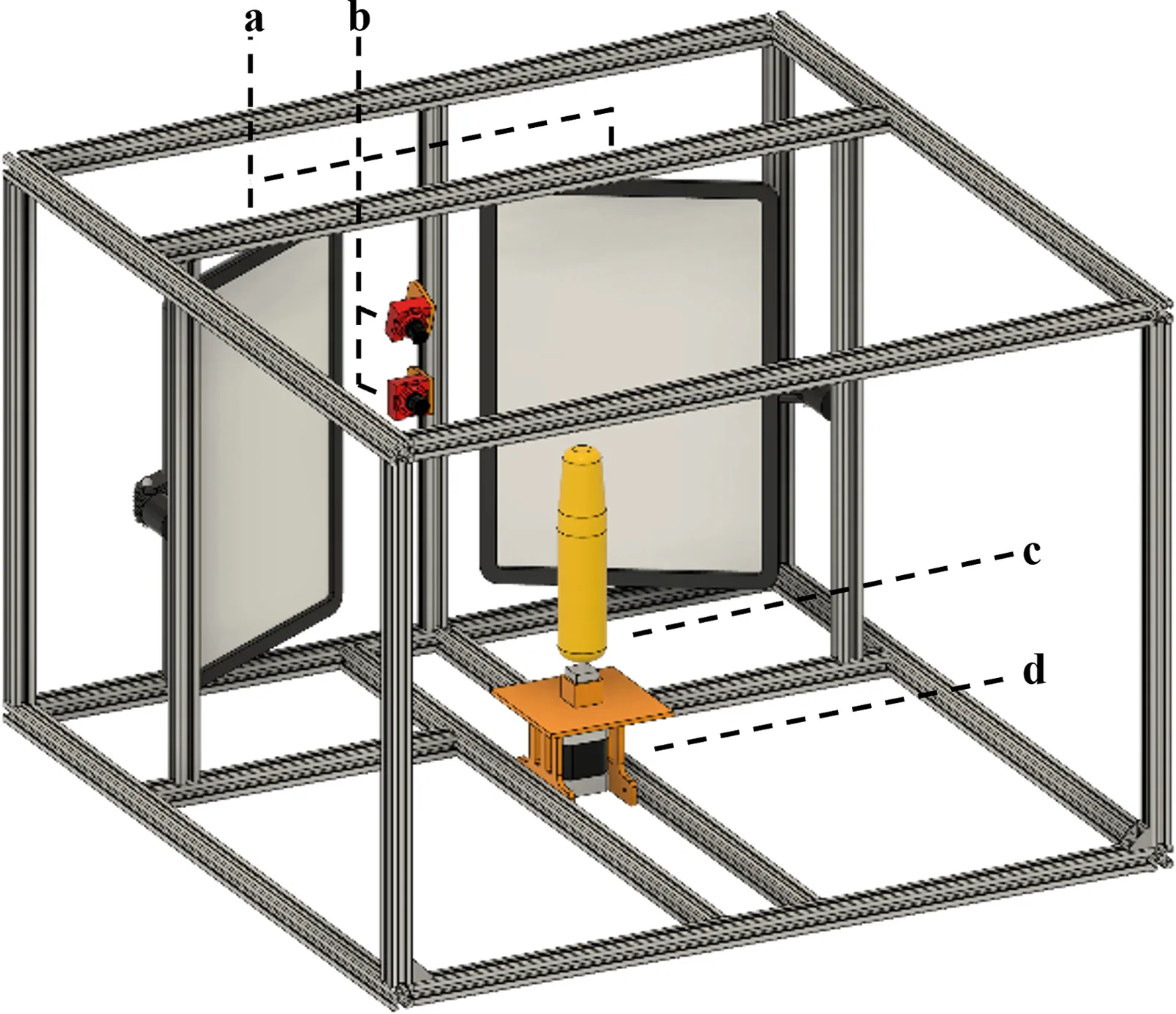
Ear scanner: (a) LED illumination panels, (b) RGB cameras, (c) maize ear mounted on a spike, and (d) stepper motor for ear rotation.

Prior to the manual measurements, all ears from both the reference dataset and the P dataset were analysed in the ear scanner. The ear scanner (Fig. 1) was constructed within an aluminium frame to provide a stable imaging environment. Uniform lighting was achieved using two LED illumination panels (Fig. 1a) positioned pointing at the maize ear. Although not shown in Fig. 1, the aluminium frame was additionally clad with white-coloured aluminium panels to ensure diffuse lighting conditions. Additionally, white paper was attached to the frame behind the ear to provide a homogenous imaging background. Two cameras featuring a resolution of 1944 x 2592 (Alvium 1500 C-500c, Allied Vision Technologies GmbH, Stadtroda, Germany) were mounted vertically one above the other (Fig. 1b). The lower camera is oriented horizontally while the upper camera is tilted downwards by *θ = *17°. This enables capturing markers below the ear's base as well as the entire ear at an EL up to 300 mm, as can be seen in Fig. A. 1. Markers were used to align camera positions relative to ear rotation. They were generated using the AprilTag library (3.4.2) [22] with the tag family *tagStandard41h12.* A single maize ear was mounted vertically on a spike (Fig. 1c) at the centre of the imaging chamber. Controlled rotation of the ear was achieved using a stepper motor (ACT-2801K, ACT MOTOR GmbH, Bremen, Germany) (Fig. 1d), which advanced the ear in increments of *ω* = 7.1°. By synchronously capturing images from both cameras and incrementing the motor 50 times, the ear was recorded from 100 distinct viewpoints. Images were acquired only when the motor was stationary to ensure image stability.

The stepper motor was driven by a microcontroller (Pico, Raspberry Pi Ltd, Cambridge, UK) powered and commanded via serial interface over USB from a microcomputer (Jetson Nano, NVIDIA Corporation, Santa Clara, USA) running on JetPack (4.4). The microcomputer also controlled the cameras via camera serial interface (CSI), ensuring proper synchronization between motor rotation and image acquisition. The responsible software on the microcontroller and the microcomputer was written in MicroPython (1.20) and Python (3.6), respectively.

The ear scanner was controlled by a single person via keyboard, mouse and monitor to start the scanning process. Each ear was mounted on a spike attached to a metal cube which is directly plugged into a platform mounted on the stepper motor, allowing for rapid placement of the ear.

### 2.4 Image processing pipeline

All training of deep learning models and image processing was done on a workstation featuring a GPU with 24 GB of VRAM (RTX 3090, NVIDIA Corporation, Santa Clara, USA), a processor with 16 cores running at 3.4 GHz (Ryzen 9 5950X, Advanced Micro Devices, Inc., Santa Clara, USA) and 128 GB of DDR4 RAM.

#### 2.4.1 Software packages

The software was implemented in Python (3.10) [23] and relies on widely used packages from the Python ecosystem. Conventional image algorithms were applied from OpenCV (4.10) [24] while Pandas (2.2.3) [25] was used for tabular data handling. Scikit-learn (1.5.2) [26] was used for statistics and clustering of kernels to predict KRN. Algorithms dealing with voxel carving were computed in JAX (0.5.0) [27]. Training and inference of the YOLO models were done in the Ultralytics framework (8.3.38) [28] which was based on PyTorch (2.5.1) [29] for deep learning utilities. Besides YOLO, the first generation segment anything model (SAM) (1.0) [13] was applied for segmentation. The TorchMetrics package and the Monai package (1.5.0) [30] were applied for segmentation and object detection metrics [31]. For statistical analysis of the P dataset, the Statsmodels package (0.14) [32] was used to compute ANOVA and the SciPy package (1.16.1) [25] was used to compute the Shapiro–Wilk test and Levene test.

#### 2.4.2 Instance segmentation experiment

To automatically detect kernels on maize ear images, different YOLOv8-seg [33] models capable of both object detection and instance segmentation were trained using the Ultralytics package. While the maximum number of epochs was set to 300, other training settings were kept at default. Masks predicted by YOLOv8n (3.2 × 10^6^ parameters), YOLOv8m (25.9 × 10^6^ parameters), and YOLOv8x (68.2 × 10^6^ parameters) were compared with masks predicted by SAM when using YOLO bounding boxes as input prompts for SAM. The smallest ViT-B encoder (91 × 10^6^ parameters) was selected for SAM owing to its computational efficiency [13].

For manual annotation, ten maize ears from the same sample pool as the reference dataset were imaged with the ear scanner. 500 raw images from the lower camera were undistorted, and only the centre crops were annotated following Oehme et al. [34], with each presumably fertile kernel being labelled as a separate instance segmentation mask. None of the ears used for annotation were present in the reference dataset or in any further experiments. Training and validation were performed on images from eight ears, with 300 images randomly selected for training and 100 images for validation. The 100 images of the remaining two ears were reserved for testing to avoid information leakage caused by overlapping image content.

The evaluation of predictions was based on mean average precision (mAP) and the dice score (DS). Ground truth polygons were rasterized into binary masks at image resolution, and bounding boxes were derived as axis-aligned rectangles. Predictions from YOLO were obtained either as polygons or bounding boxes with associated confidence scores, whereas SAM produced binary masks that were assigned a default confidence of 1.0 and a class label of 0. All predictions were matched against the ground truth following the COCO evaluation protocol [35].

For mAP computation, TorchMetrics using the *faster_coco_eval* backend was applied with intersection over union (IoU) thresholds ranging from 0.50 to 0.95 in steps of 0.05. Thus, the highest-confidence predictions were assigned first to the ground truth masks with the highest IoU. Each ground truth mask was matched at most once, ensuring that subsequent lower-confidence predictions could only be paired with the remaining unmatched objects. A match was considered correct if the IoU exceeded the threshold, otherwise the prediction was counted as a false positive, while unmatched ground truth instances were considered false negatives. Precision–recall curves were then constructed by sweeping the confidence threshold across the ranked predictions, and the average precision was obtained as the area under the curve. mAP was defined as the mean of these values across IoU thresholds, with separate results reported for bounding boxes, YOLO masks, and SAM masks.

The formula for mAP in the given single class scenario can be written as

(2) $$\begin{array}{c} mAP=\frac{1}{|T|}\sum_{\tau \in T}\int_{0}^{1}p_{\tau }\left(Recall\right)dr\text{,} \end{array}$$

where = {0.50,0.55, …, 0.95}is the set of IoU thresholds for mAP_50-95_, while = {0.50} for mAP_50_ and = {0.75} for mAP_75_. *p*_*τ*_*(Recall)* is the enveloped precision as a function of recall at IoU threshold *τ*. In the case of bounding boxes, the IoU was computed between axis-aligned rectangles, whereas for segmentation masks it was computed between binary mask regions.

In addition, the DS was computed using the MONAI package. Unlike mAP, which assesses instance-level detection and localization, the DS captures how accurately the overall segmentation aligns with the ground truth across the entire image.

Here, the DS is

(3) $$\begin{array}{c} DS=\frac{2\left|A\cap B\right|}{\left(\left|A\right|+\left|B\right|\right)}\text{,} \end{array}$$

where *A* and *B* denote the sets of pixels belonging to the predicted and ground truth masks, respectively. A∩B thus represents the overlapping area between both sets.

#### 2.4.3 Digital trait measurements

This study aims to replace the manual measurement of multiple maize ear traits by measurements based on RGB images. Therefore, camera extrinsic parameters were estimated relative to a fixed ear-centred coordinate system using AprilTags. At least three fiducial markers were detected in each image, and their 2D pixel coordinates were matched to corresponding 3D reference positions from a calibrated marker layout, as can be seen in Fig. A. 1. Using the known distorted camera intrinsics *K*_*d*_, the perspective-n-point (PnP) problem was solved in OpenCV to minimize reprojection error, yielding the rotation vector *r* and translation vector *t*. These were converted to a rotation matrix *R* and combined into the 4×4 extrinsic transformation matrix [*R*∣*t*]. The resulting pose defines the camera's position *C=−R*^*T*^*t* in world coordinates.

**Fig. 2:**
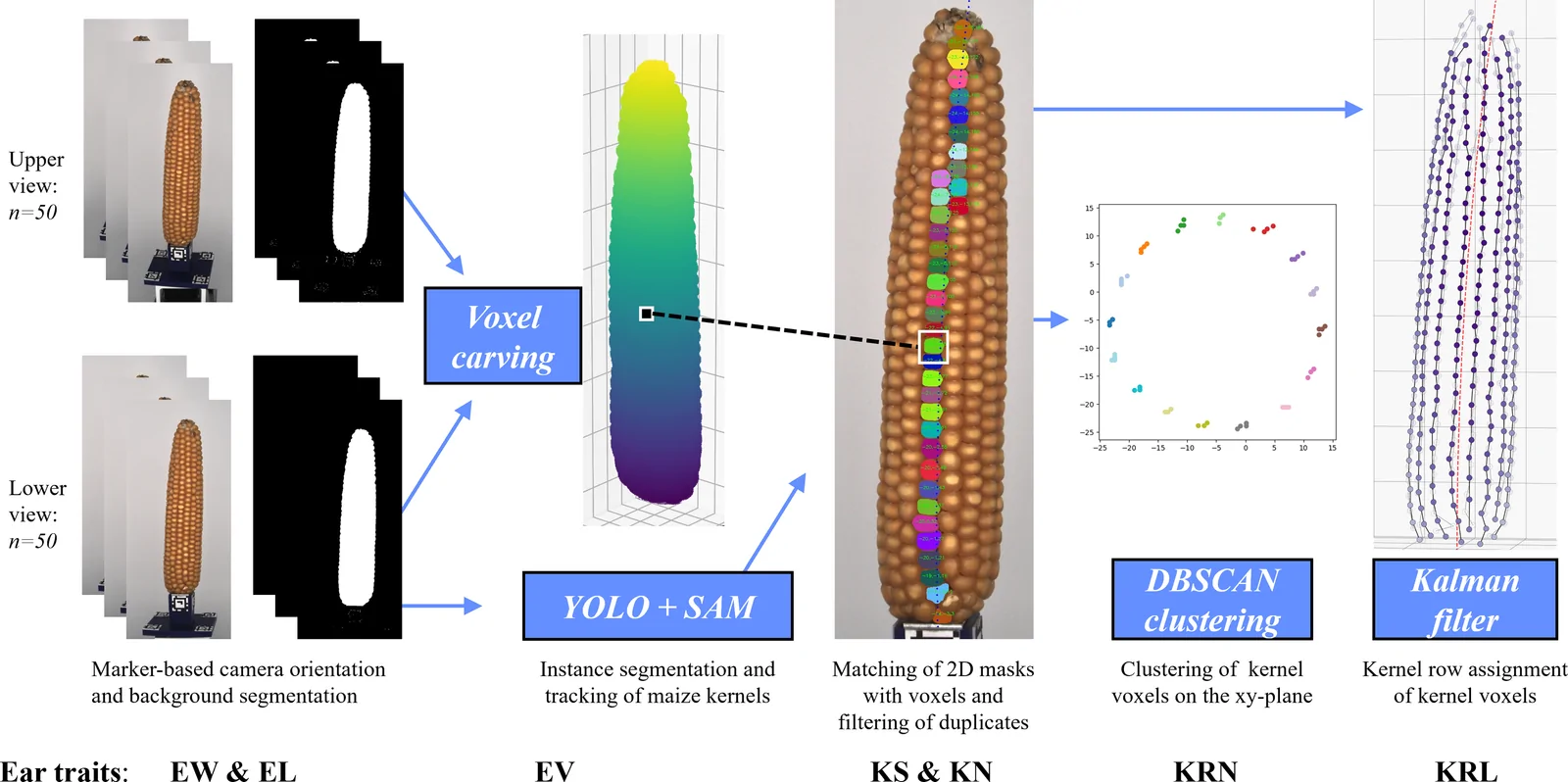
Processing workflow of digital ear trait measurements. Key algorithms are displayed in blue boxes. Ear traits are shown in bold letters below the process step in which they were computed. EW: Ear width; EL: Ear length; EV: Ear volume; KS; Kernel size; KN: Kernel number; KRN: Kernel row number; KRL: Maximum kernel row length.

For all digital trait measurements, raw images were undistorted using the camera intrinsics, thereby removing radial and tangential lens distortions. This correction yielded a uniform spatial scale, permitting direct conversion from pixel dimensions to physical units (mm) on planes approximately orthogonal to the camera's optical axis. Since the lower camera was positioned horizontally, EL and EW were subsequently extracted from its undistorted images. Here, ear silhouettes were derived from the undistorted images by thresholding in the HSV colour space (H ∈ [0, 38], S ∈ [55, 255], V ∈ [0, 255]) to separate the ear from the background (Fig. 2). The resulting binary mask was used to extract the ear contour. A minimum-area bounding rectangle was then fitted to the ear contour in each of the 50 images captured by the lower camera. The median values of the rectangles' shorter and longer sides were defined as EW and EL, respectively.

Voxel carving as shown in Ref. [36] was applied to integrate all 100 ear images into a coherent 3D model, enabling the translation of 2D image features into 3D space. A 3D voxel grid was initialized over the ear crop bounds as previously defined by the maximum ear silhouette dimension, which were retrieved over all minimum-area bounding rectangles (Fig. 2). Similarly to the silhouettes of the lower camera, silhouettes for the upper camera were computed, allowing precise voxel carving from all 100 views. Consequently, tilted and large ears require a larger voxel grid while smaller and straight ears require a smaller one. The resolution of the voxel grid was set to 0.5 mm length per voxel side, providing a balance between spatial resolution and computational efficiency. Using the undistorted camera intrinsics *K*_*u*_ and the extrinsic matrix [*R*∣*t*], camera projection matrices *P*=*K*_*u*_[*R*∣*t*] were computed for each image view. During voxel carving, each voxel centre was projected into all camera views via its projection matrix. A voxel was retained only if its 2D projection lied within the ear silhouettes in all images. Voxels failing this condition were discarded. Finally, EV was obtained by counting the number of remaining voxels.

The detection of KN per ear was based on the *m*-weights of the YOLOv8 detection model [37] and was only done on images of the lower camera. To avoid duplicate detections, the kernels were tracked over all 50 images using Ultralytics' track functionality kept at default parameters, which is based on BoT-SORT [38]. Since more than 100 kernel instances were often detected in a single view of a large ear, the input ear images of ears with width ≥ 42 mm were cropped to the central 60% of the view's minimal area bounding box width, thus omitting irrelevant side view kernels. While this approach facilitates tracking only relevant kernel instances on wider ears, it also leads to limiting available kernel views on thinner ears. Therefore, images of ears thinner than 42 mm, roughly representing the mean width of the testcross population, were not cropped down to the horizontal centre. Consequently, input images of varying dimensions were resized to 640 ∗ 640 pixels for processing with YOLOv8m.

Valid kernel detections were collected as soon as a kernel was detected on the ear's central axis to avoid unreliable detections on the ear edges (Fig. 2). The central axis was derived from a parametric quadratic curve fitted through the carved voxel grid, which was computed previously. The curve is defined as

(4) $$\begin{array}{c} x\left(t\right)=a{\cdot t}^{2}+b\cdot t+c,\\ y\left(t\right)=d\cdot t^{2}+e\cdot t+f\text{,}\\ z\left(t\right)=t\;\text{.} \end{array}$$

Here, *x(t)*, *y(t)* and *z(t)* represent the 3D coordinates of the curve at parameter *t*, which equals the z-coordinate. The coefficients *a, b, c, d, e* and *f* are obtained by minimizing the squared fitting error using least-squares regression. Since detections were only retrieved from this optimal centre view, no further matching or modification of detections was considered beneficial for detection quality.

After running tracked object detection on the lower camera's images of a given ear, the centre of each bounding box was mapped into 3D by applying the projection matrix *P* for the corresponding image (Fig. 2). This step enabled the identification and removal of duplicate detections, particularly those occurring in the first and last images, as well as kernels that were incorrectly tracked. Duplicate kernels were defined as those with 3D positions separated by less than 2 mm in an anisotropically scaled coordinate system. Duplicate filtering proceeded iteratively in the order in which the images were acquired. Kernels detected earlier in the sequence were retained, while later detections falling within the threshold distance were discarded. Before computing distances, the *x* and *y* coordinates of both the current voxel candidates and the previously accepted kernel positions were scaled by a factor of 0.5. This anisotropic scaling reduced the influence of horizontal displacement relative to vertical displacement and improved robustness against small lateral tracking errors.

The bounding boxes corresponding to the remaining kernels were subsequently used as input prompts to generate segmentation masks with SAM. Individual kernel size was calculated by converting the number of pixels in each kernel mask into mm^2^. The conversion scale was obtained from the distance between the camera and the voxel corresponding to each kernel. Subsequently, the resulting kernel sizes were averaged for each ear to retrieve the KS. Similarly, the bounding box width (*x*-axis) and length (*y*-axis) were scaled from pixels to mm and averaged per ear to compute mean box width (BW) and mean box height (BH), respectively.

The previously computed 3D kernel positions were also used to predict the KRN (Fig. 2). First, the vertical extent of the ear was quantified using the minimum and maximum *z*-coordinates across all kernels, and a central slab spanning the middle 10% of this range was defined to obtain a representative cross-section of kernels. Kernel positions within this slab were projected into the *xy*-plane, and the resulting 2D coordinates were clustered using DBSCAN [39] with an epsilon value of 3 mm and a minimum cluster size of two points. The number of retained clusters was interpreted as the number of kernel rows. Because the biological arrangement of maize kernels produces typically an even number of rows, an uneven number of detected kernel rows was rounded up to the next even number.

To assign every detected kernel to a kernel row (Fig. 2), it was assumed that kernel rows follow a roughly consistent trajectory which is roughly parallel to the central ear axis in Eq. (4). Therefore, two Kalman filters [40] were initialized per kernel, with one predicting the upward direction and another one predicting the downward direction. For simplicity, no control input that would compensate for external drivers affecting the kernel row trajectories was used. Thus, each Kalman filter follows

(5) $$\begin{array}{c} x_{k}=F\;x_{k-1}+w_{k}\text{,} \end{array}$$

where *x*_*k*_ = [*x, y, z, v*_*x*_*, v*_*y*_*, v*_*z*_]^*T*^ is the state vector containing 3D position and velocity of the kernel.

*F* is the constant-velocity state transition model, and *w*_*k*_ ∼ *N*(0, *Q*) is Gaussian process noise.

After initialization, the Kalman filter iteratively predicts the subsequent kernel position, matches this prediction with an actual kernel position, updates its state estimate based on the resulting innovation and then proceeds to predict the next position. Matching of predicted kernel positions with actual candidate kernels is performed using Mahalanobis distance [41] with a chi-square gating threshold (*p* = 0.99). If no matches could be found lying within the gate, the iteration terminates and the kernel row is assumed to have reached an end. Normally, these ends would represent the uppermost or lowermost kernel of a row.

However, if a kernel was matched, the filter is updated using

(6) $$\begin{array}{c} z_{k}=H\;x_{k}+v_{k}\text{,} \end{array}$$

where *z*_*k*_ is the observed 3D kernel position, *H* is mapping the actual kernel position into the predicted position, and v_k_ ∼ *N*(0, *R*) is measurement noise.

Each kernel connection that was detected based on the Kalman filter procedure was given a matching score

(7) $$\begin{array}{c} score=1-\frac{\sqrt{d_{\min}^{2}}}{\sqrt{T}}\text{,} \end{array}$$

where *d*^*2*^_*min*_ is the squared Mahalanobis distance [41] and *T* is the chi-square gating threshold. This score is summed for each kernel connection over all Kalman filter iterations where the same kernels were connected. Since each kernel can only be part of one kernel row, each kernel can have at maximum one connection upwards and one connection downwards. If the Kalman filter found more than one connection per upward or downward direction, the lowest ranking connections according to the matching score were pruned. After this step, each kernel had at most two kernel connections. However, the pruning step could result in single kernels and incomplete kernel row snippets. In a subsequent postprocessing step, kernel row endpoints and single kernels were identified to be matched with other endpoints or single kernels within a distance of twice the previously computed average kernel height. Due to the small abundance of possible matches after most kernels have already been assigned to a kernel row, this matching procedure is intended to search in a larger space than the matching of the Kalman filter predictions. Thereby even matches are possible if a kernel is missing in a row. Finally, the predicted KRL is defined as the maximum length of the detected kernel rows.

### 2.5 Statistical analysis

For each trait examined in the P dataset, treatment effects were assessed using a linear model including repetition block from the field design as fixed factor. The model was fitted using ordinary least squares (OLS) and significance between treatments was evaluated using a Type II ANOVA. Assumptions of normality and homogeneity of variances were evaluated using the Shapiro–Wilk and Levene tests, respectively. Due to a robust sample size of 44 ears from the unfertilized control and 45 ears from the fertilized treatment a stricter significance threshold (α = 0.01) was applied for both tests. Traits that met both assumptions were analysed using ANOVA.

## 3 Results

### 3.1 Instance segmentation of maize kernels

**Fig. 3:**
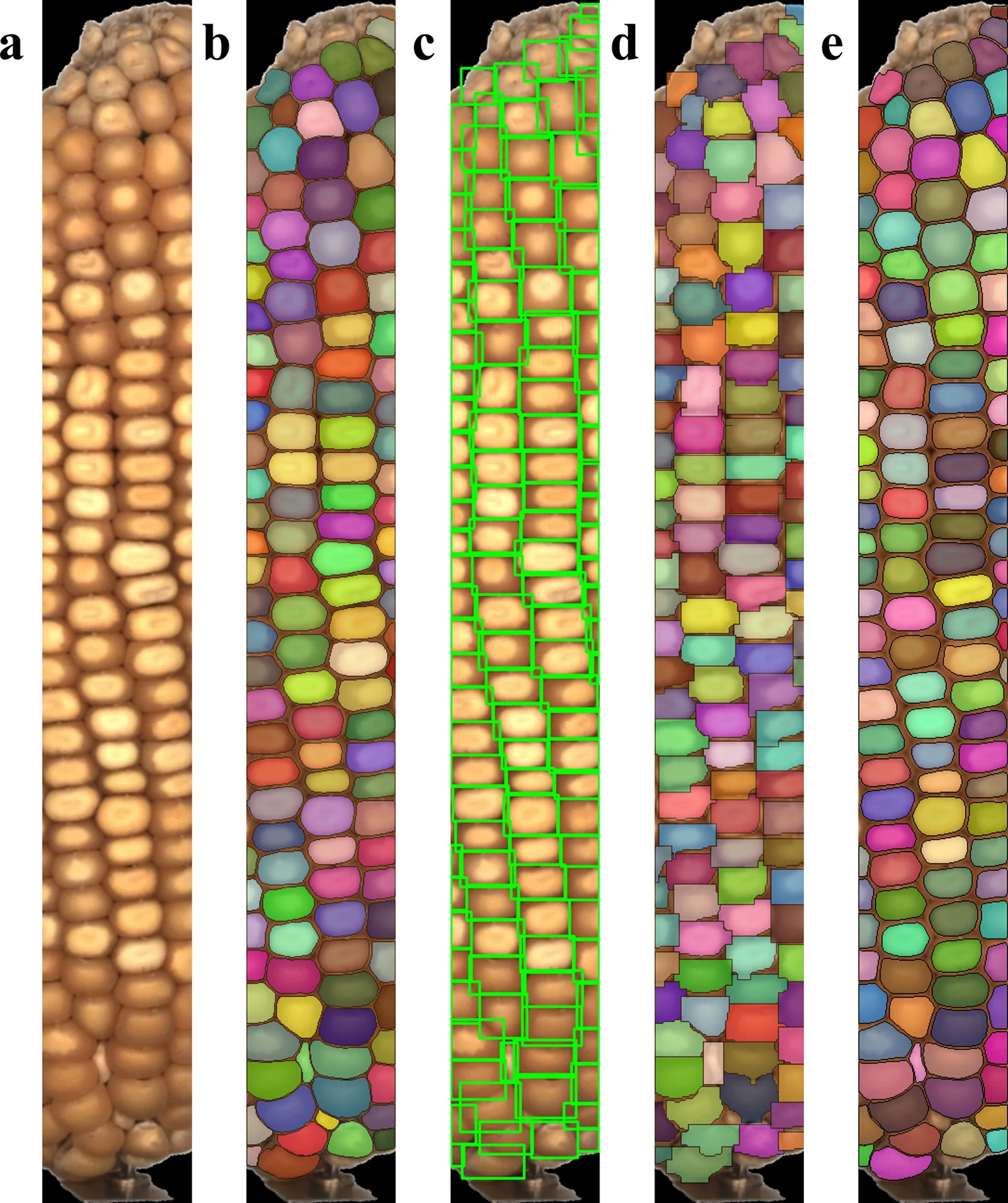
Maize kernel detection on centre ear images captured by the ear scanner. (a) RGB image. (b) Ground truth masks. (c) Bounding box predictions by YOLOv8m. (d) Segmentation mask predictions by YOLOv8m. (e) Segmentation mask predictions by SAM after prompting boxes from (c). All masks are depicted in random colours.

Each ear was imaged from 100 viewpoints, collected within 30 s by the here presented ear scanner. To establish an instance segmentation model capable of detecting kernels, different YOLOv8 variants were finetuned and compared on crops of the lower camera's images (Fig. 3a). Table 1 shows mAP_50-95_ results of the YOLOv8 models on both object detection (bounding boxes) and instance segmentation (masks) capabilities. Here, performance consistently increased with model size. YOLOv8x achieved the highest detection accuracy with an mAP_50-95_ of 0.849, followed by YOLOv8m and YOLOv8n. A similar trend was observed for instance segmentation, although the improvements in mask mAP were relatively modest across variants (0.499–0.507).

**Table 1:** Performance comparison of YOLOv8 model variants across object detection and instance segmentation tasks. Predicted bounding boxes from YOLO were used as prompt input to create SAM masks. The best performance per column is highlighted by bold numbers. mAP: Mean average precision, DS: Dice score.

Weights	YOLO boxes	YOLO masks		SAM masks	
	mAP_50-95_	mAP_50-95_	DS	mAP_50-95_	DS
YOLOv8n	0.8224	0.4991	0.9342	0.7063	0.9391
YOLOv8m	0.8358	0.5038	**0.9369**	**0.7198**	**0.9401**
YOLOv8x	**0.8485**	**0.5073**	0.9360	0.7184	0.9398

In contrast, the DS of the YOLO-generated masks remained uniformly high (>0.93), indicating that even the smallest model produced spatially accurate segmentation outlines once an object was detected. When using YOLO-predicted bounding boxes as prompts for SAM, segmentation performance further improved, reaching DS of 0.939–0.940 across all weights. This demonstrates that SAM can reliably refine object boundaries independent of the underlying YOLO variant. However, the YOLOv8m weight in combination with SAM achieved the highest mAP_50-95_ over all segmentation variants (0.720). Thus, YOLOv8m prompting bounding boxes for SAM has been applied for instance segmentation in all further experiments of this study. Predictions on a random image of the test set can be seen in Fig. 3. Here, predicted bounding boxes (c) and masks (d) from YOLOv8m as well as predicted masks from SAM (e) are compared to ground truth masks (b). Masks of SAM closely reassemble the ground truth masks while the YOLOv8m masks appear less accurate, which is in accordance with the results shown in Table 1. However, SAM appears to slightly over-segment kernels compared to the ground truth annotation.

### 3.2 Establishing digital trait measurements based on the ear scanner

**Fig. 4:**
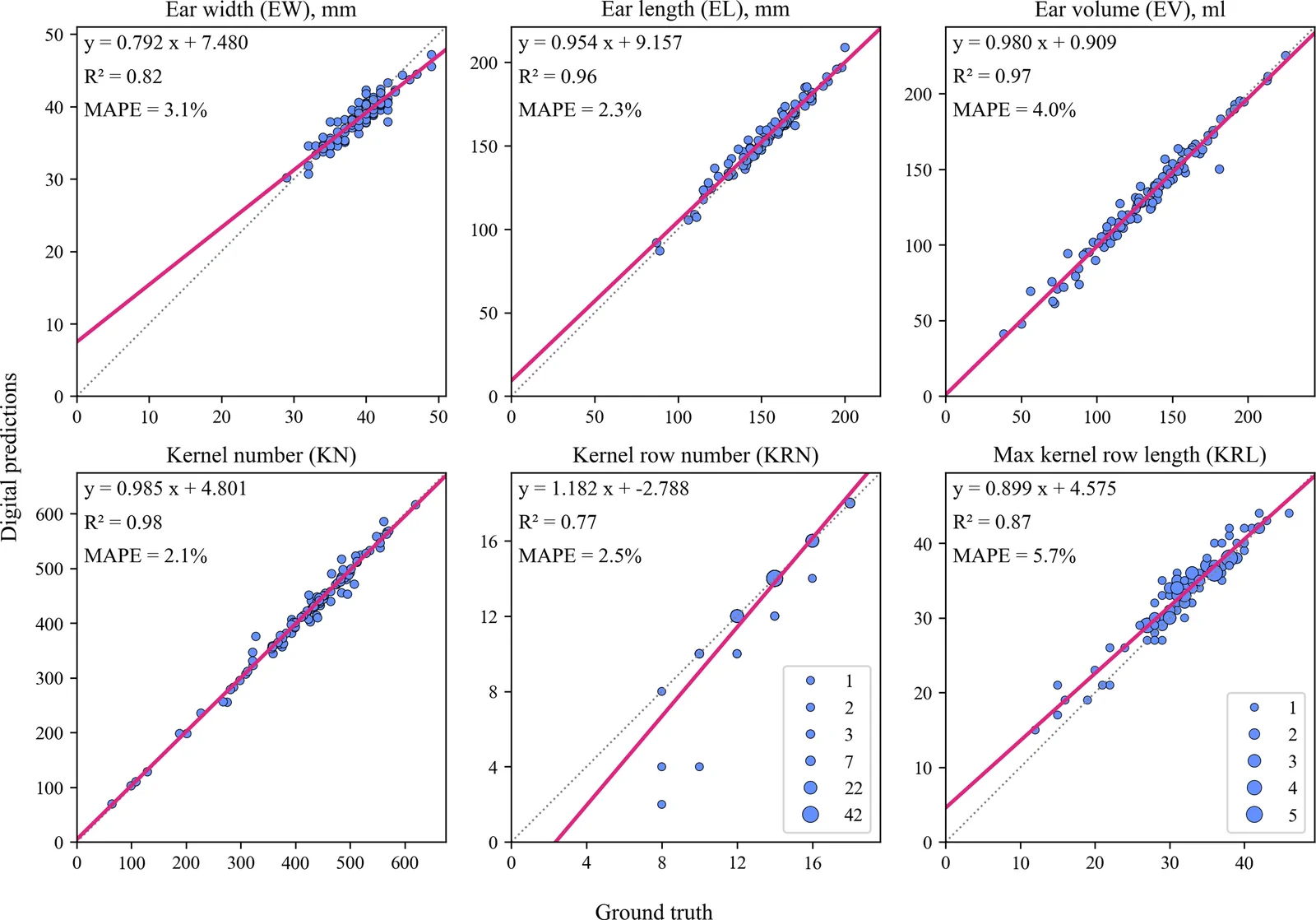
Comparison of manual ground truth measurements (abscissa) and digital predictions (ordinate) based on the ear scanning pipeline. The grey dotted line in each plot indicates 1:1 relation while the red line is the fitted linear regression. Equations describing the regression models, the *R*^*2*^ score and mean average percentage error (MAPE) are displayed in the top left corner of each plot. In the two bottom-right plots, point size indicates the number of overlapping data points.

Multiple ear traits have been measured manually on all 105 maize ears of the reference dataset. Across the dataset, most traits showed considerable variation. EL averaged 152.2 ± 22.5 mm (mean ± standard deviation), EV 132.8 ± 35.5 ml, KN 419.5 ± 103.9, KRL 32.1 ± 6.1 and KRN 13.9 ± 2.1, all indicating relatively wide dispersion. In contrast, EW (39.1 ± 3.6 mm) exhibited comparatively low standard deviations. This also becomes obvious when looking at Fig. 4, where ground truth measurements are shown along the *x*-axes. While EW data points cover only a small portion of the x-axis, all other traits exhibit a broader distribution along the x-axis.

To compare the manually measured ground truth to the digital predictions, the same ears were captured by the ear scanner resulting in 10,500 images. Following the capturing procedure, the image processing pipeline took on average 47s to process the 100 images per ear. One of the initial steps of the pipeline is to predict the EW and EL. As can be seen in Fig. 4, both traits can be predicted with very small MAPE of 3.1% and 2.3%, respectively, when compared to manual reference measurements. This corresponds to an R^2^ of 0.82 for EW and an R^2^ of 0.96 for EL, indicating a close agreement between predicted and reference values.

**Fig. 5:**
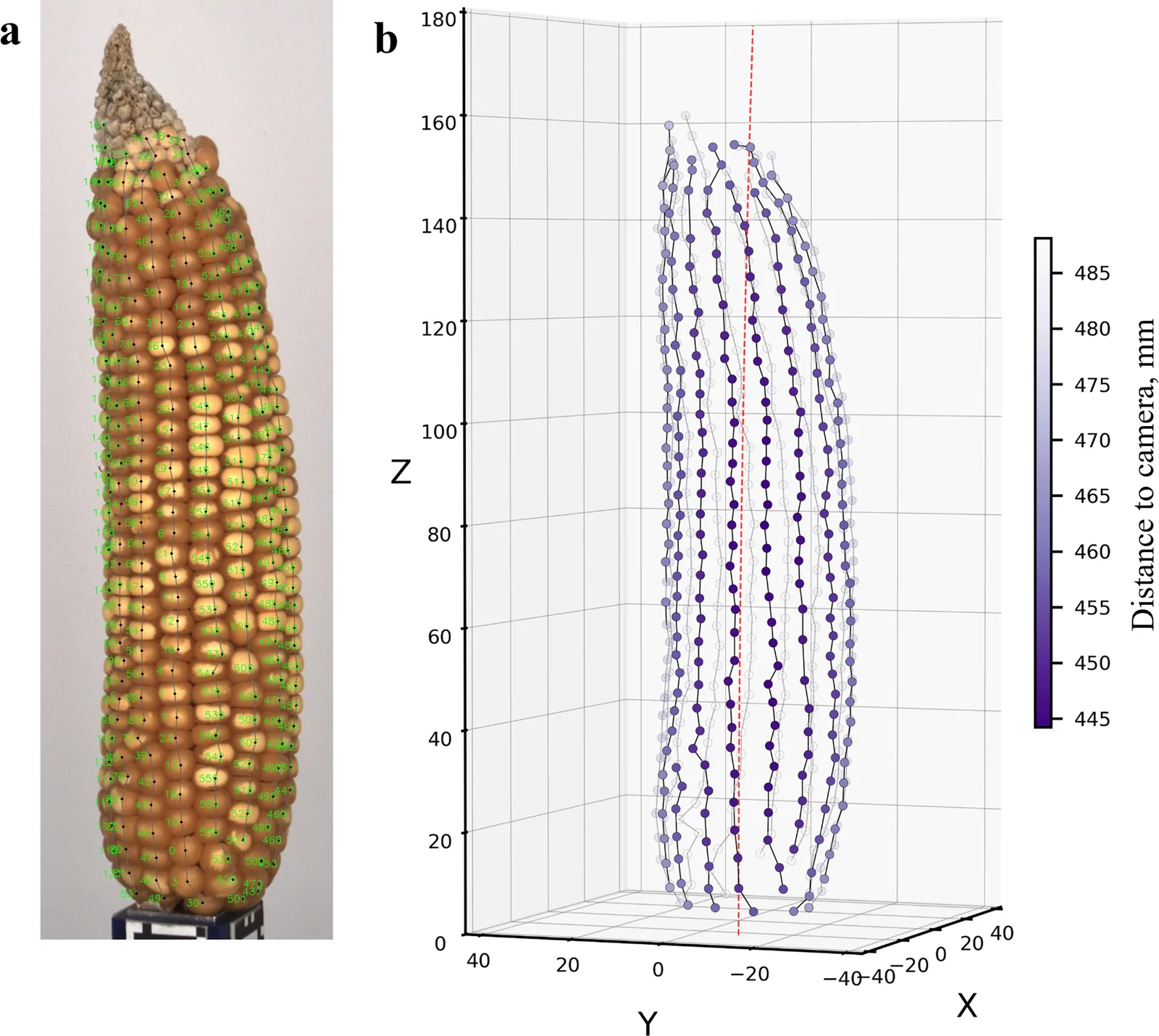
3D-oriented maize kernel detections. (a) Undistorted RGB image with kernel positions indicated by green ID numbers and black points. Grey lines indicate detected kernel rows. (b) Maize kernel detections in 3D space from the same view as (a). The colour of points represents the distance of the detected kernel to the lower camera while members of the same kernel row are connected with black lines. The red dotted line indicates the predicted ear centre.

The subsequently processed EV was estimated based on voxel carving with an R^2^ of 0.97 and a MAPE of 4.0% (Fig. 4). KN exhibited the strongest overall performance with an R^2^ of 0.98 and a MAPE of 2.1%. The high prediction accuracy for EV and KN demonstrates that the tracking and 3D orientation of individual kernels worked reliably. Fig. 5a shows the unique kernel IDs (green colour) that can be projected back on every original RGB image to proof successful tracking of maize kernels.

KRN showed a slightly lower R^2^ of 0.77, but still a very low MAPE of 2.5%. The KRL was predicted with an R^2^ of 0.87, an RMSE of 2.18, and a MAPE of 5.7% (Fig. 4). Successful assignment of kernels into kernel rows is also displayed in Fig. 5, displayed as lines connecting individual kernels. The red dotted line in Fig. 5b indicates the detected ear centre, which serves as base to compute the unit vector from which the initial kernel row direction is assumed as described in the method section. At the given view in Fig. 5 the kernel rows appear roughly parallel to the ear centre curve, suggesting that the curve derived unit vector is a good indicator for the direction a kernel row takes.

The predicted segmentation masks of all kernels of this ear can be seen in Fig. A. 2, where the masks are arranged in the detected kernel rows. Together, the reported metrics demonstrate that the proposed pipeline provides accurate, high-throughput digital phenotyping of ear morphological traits.

### 3.3 Identifying specific ear traits in response to P fertilization

After establishing and evaluating the ear-scanning pipeline for accurate extraction of multiple traits, the method was applied to ears from a P fertilization experiment to demonstrate the method's applicability. A total of 89 ears, 44 ears of the unfertilized control and 45 ears of the P treatment, were scanned.

**Fig. 6:**
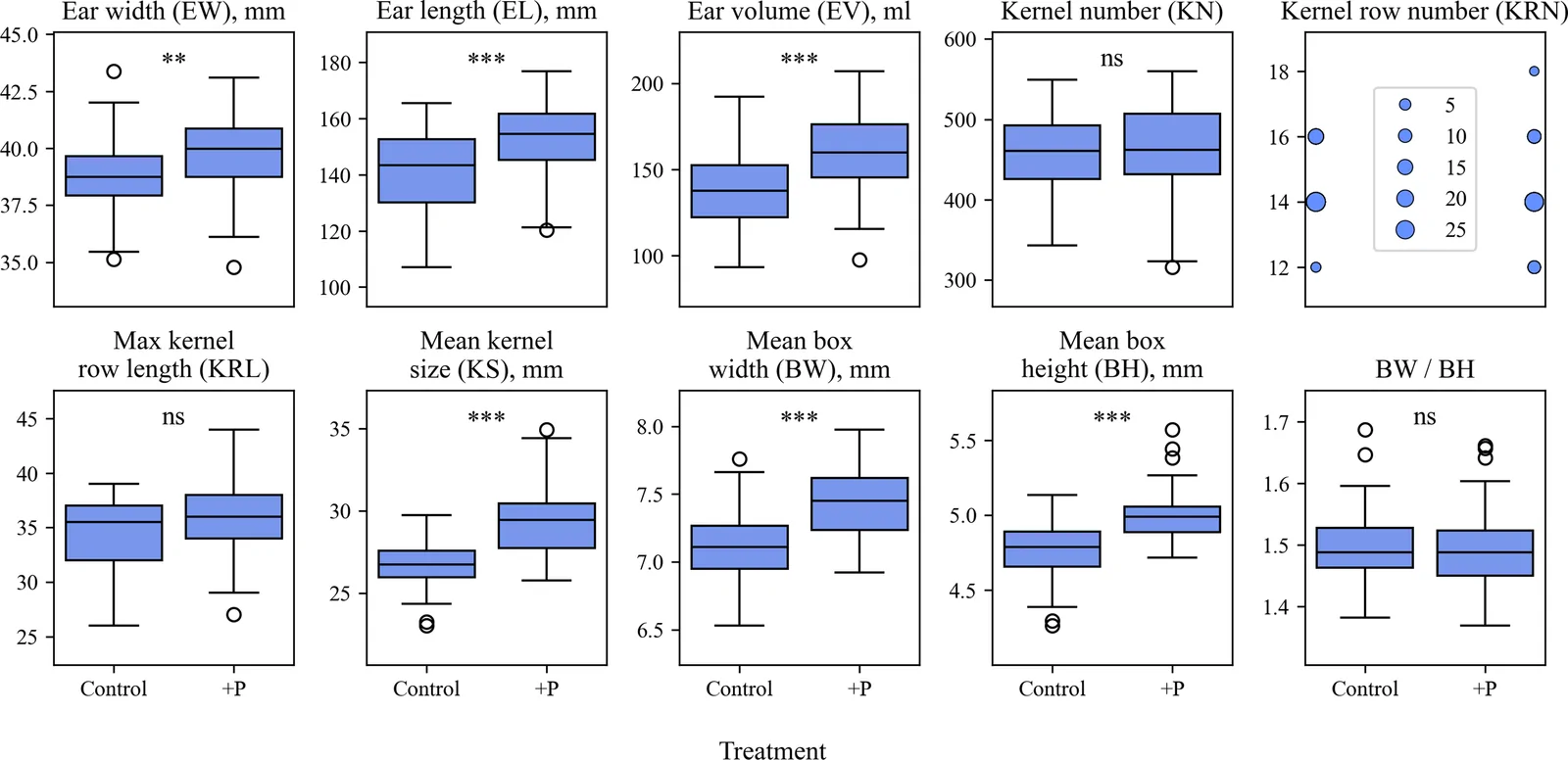
Comparison of maize ear traits between unfertilized control plants and plants receiving P fertilizer. In the kernel row number panel, circle sizes correspond to the frequency of observations at each y-value per treatment. ANOVA was conducted for all other traits, and significance levels are indicated between boxplots (ns: p ≥ 0.05; ∗: p < 0.05; ∗∗: p < 0.01; ∗∗∗: p < 0.001).

As can be seen in Fig. 6, applying the ear-scanning pipeline to the P-trial revealed that several traits responded significantly to P-supply. Fertilized plants showed slightly wider EW (39.8 ± 1.8 mm) than the control group (38.7 ± 1.7 mm; F_1_,_83_ = 9.0, p = 0.004). EL and EV showed a strong response to fertilization, rising from 141.0 ± 15.3 mm to 153.0 ± 13.3 mm (F_1_,_83_ = 16.5, p < 0.001) and from 137.2 ± 22.9 ml to 159.0 ± 22.8 ml (F_1_,_83_ = 22.6, p < 0.001), respectively. Kernel morphology traits exhibited similar treatment responses with KS increasing from 26.7 ± 1.4 mm^2^ in the control to 29.3 ± 1.9 mm^2^ under P fertilization (F_1_,_83_ = 56.1, p < 0.001), while the related BW and BH were also significantly larger in fertilized plants (7.1 ± 0.3 mm vs. 7.5 ± 0.3 mm; F_1_,_83_ = 35.1, p < 0.001 and 4.8 ± 0.2 mm vs. 5.0 ± 0.2 mm; F_1_,_83_ = 38.4, p < 0.001 respectively).

In contrast, several other traits showed no significant fertilization effect. KN did not differ between treatments (458 ± 49 vs. 463 ± 54; F_1_,_83_ = 0.2, p = 0.67), nor did maximum KRL (34.7 ± 3.4 vs. 35.7 ± 3.4; F_1_,_83_ = 1.9, p = 0.17), or the BH to BW ratio (1.5 ± 0.1 in both treatments; F_1_,_83_ ≈ 0, p = 0.96). Since normality could not be assumed for KRN, there was no ANOVA computed. Judging from the mean and standard deviation of the control (14.6 ± 1.1) and P treatment (14.2 ± 1.4), no relevant P response would be expected for KRN.

## 4 Discussion

### 4.1 Instance segmentation of maize kernels

In this study, multiple kernel related traits as predicted by the ear scanner pipeline have been established. The base of measuring kernel traits was to train and to implement a deep learning approach capable of accurate instance segmentation. Here, YOLOv8m prompting bounding boxes to SAM was identified as the most accurate approach (Table 1 and Fig. 3). This combination of a fine-tuned model for object detection (YOLOv8m) and direct application of a foundation model for instance segmentation (SAM) only requires training of the object detection model. Moreover, the training of an object detection model requires only annotation of bounding boxes instead of more time-consuming segmentation masks. This strategy allows for saving costs on both human labour for the annotation of training data and on computation time for AI-training.

Notably, the instance segmentation test set only consisted of 100 images originating from two ears, which cannot be considered a diverse set of phenotypes. However, while the model was only trained and validated on eight other ears, the two unseen test ears could even represent a small domain gap, thus demonstrating the model's robustness. Additionally, the model contributed to accurate predictions of KN, KRN and KRL on the morphologically diverse ear trait reference set, which underlines its robustness on previously unseen data.

The requirement to have loaded two deep learning models (YOLOv8m and SAM) simultaneously into GPU for efficient computation poses high demands on VRAM, which was satisfied by the RTX 3090 with 24 GB of VRAM. To be able to run the processing pipeline on lower cost hardware or to accelerate the processing speed, it would be more efficient to run a single model for instance segmentation. The low segmentation quality of YOLOv8m that can be seen in Fig. 3d could be improved by feeding the image as downsized crops to the model instead of the whole image at once. Here, images of ears with high EL often exhibited more than 1500 pixels on the y-axis and had to be downsized to 640 pixels as required by the standard implementation of YOLOv8m [28]. In contrast, SAM accepts an input image size of 1024 pixels per axis [13], thus the loss of image information due to down-sampling appears to be less critical. Independent of the downsizing problem, SAM would be expected to perform better on segmentation tasks than YOLOv8m due to its size (YOLOv8m (25.9 × 10^6^ parameters) vs. SAM ViT-B encoder (91 × 10^6^ parameters)) and being trained specifically for segmentation tasks. However, even more computation efficient SAM encoders like those of SAM 2 [42] could be integrated in the existing pipeline and accelerate the processing.

Additionally, approaches like SAHI [43] could split the larger images into small overlapping tiles, feed them to the model and return the detections oriented to the original image dimensions. Here, multiple inference runs of the model would be required but only the YOLOv8m would be loaded into VRAM, posing lower demands on hardware. By not losing any image information with this approach, higher segmentation quality of YOLOv8m, independently of SAM, could be expected. Furthermore, if precise estimation of KS is of less interest, research groups could use the segmentation masks of YOLOv8m directly. Additionally, voxel carving allows to flexibly reduce the voxel grid resolution down to the available computation hardware.

### 4.2 Mapping 2D instance segmentation to 3D ear traits

Many approaches in 3D plant phenotyping use computational efficient and easily accessible 2D foundation models or standard model architectures and map the resulting predictions onto 3D reconstructions. These approaches differ substantially in their 3D reconstruction algorithms and mapping strategies. Feature descriptor-based algorithms like structure from motion (SfM) and multi-view stereo (MVS) have been successfully applied for instance by Santos et al. [44], who detected and tracked wine grape clusters by means of Mask R-CNN and SfM, or Wang and Zhang [45], who segmented peanut leaves with HQ-SAM based on 3D reconstruction enabled by SfM and MVS. A drawback of the common SfM-MVS pipeline is the computational demand especially for processing large image collections [46]. This problem can e.g. be overcome by employing a binocular camera, capturing a 3D point cloud and two RGB images simultaneously, as demonstrated on tomato plants by Zhou et al. [47]. However, binocular camera systems are more expensive than individual RGB cameras and only produce point clouds based on single views.

Moreover, SfM-MVS pipelines suffer from partly specular or weakly textured surfaces, due to its reliance on cross-view appearance correspondences. To tackle this issue, Doi et al. [48] proposed object instance-wise 3D reconstruction based on epipolar geometry which is independent of feature descriptors. However, this approach suffers from contact and occlusions of detected objects. Another type of 3D reconstruction, that is robust against weakly textured surfaces, is voxel carving. Das Choudhury et al. [49] successfully demonstrated voxel carving on maize for phenotyping of plant architecture, despite plant leaves showing a reflective surface.

Besides reflective kernel surfaces, special challenges of maize ear phenotyping are the high number of closely arranged kernels and the occlusion of kernel instances on ∼50% of the views. Therefore, the novel here proposed mapping of 2D instance segmentation to 3D ear traits is based on voxel grids, representing 3D reconstructions of maize ears. Similarly, in Shi et al. [50] tomato seedlings were segmented in 2D and matched to voxel-grid point clouds. Majority voting improved segmentation but was failing under occlusion. Unlike thin leaves, which require dense point correspondence for accurate area estimation, maize kernels are approximately spherical and small. Matching each 2D kernel mask to a single voxel-grid centre point is therefore sufficient for scaling ear traits measured on 2D images to real-world units and avoids the occlusion problem. This approach contributes to the computational efficiency of the here presented phenotyping pipeline, particularly given the large number of kernel instances that needed to be tracked.

### 4.3 Evaluation of predicted maize ear traits

The ear scanning pipeline achieved very high accuracy for predicting EL, KN and EV when compared to manual reference measurements (Fig. 4). This is especially remarkable for both KN and EV, since these represent traits that are time-consuming and challenging to measure manually. Regarding KN, on average 419.5 kernels per ear were manually counted for the reference set. To avoid counting kernels more than once, kernels were marked, making the method destructive. Painted kernels might not be useable for further lab analysis, colour measurements or human consumption. Similarly, manually measuring EV with the water submergence method is time-consuming and destructive. The wet conditions could trigger germination of the kernels or promote fungal diseases. In contrast all traits measured by the ear scanning pipeline are retrieved non-destructively. Only a small hole is punched into the cob's base by the spike holding the ear, which has no influence on any other standard analysis of maize ears.

According to the R^2^ values shown in Fig. 4, the ear scanning pipeline showed lower but still robust accuracy for predicting EW, KRN and KRL. Of these traits, KRL poses special challenges to manual phenotyping as it requires the observer to have an overview over the entire ear to be able to judge which kernel row has the most kernels. Typically, this was done by starting from the uppermost valid kernel at the ear tip and then progressing down the row towards the ear base. This approach can lead to identifying the wrong row as the longest if the kernels are arranged irregularly or if the ear has an incomplete kernel set. Such ears often have a relatively low KRL. Accordingly, as indicated by the regression line (*y = *0.899 *x +* 4.575) in Fig. 4, the ear scanning pipeline tends to overestimate the KRL of ears with lower KRL.

**Fig. 7:**
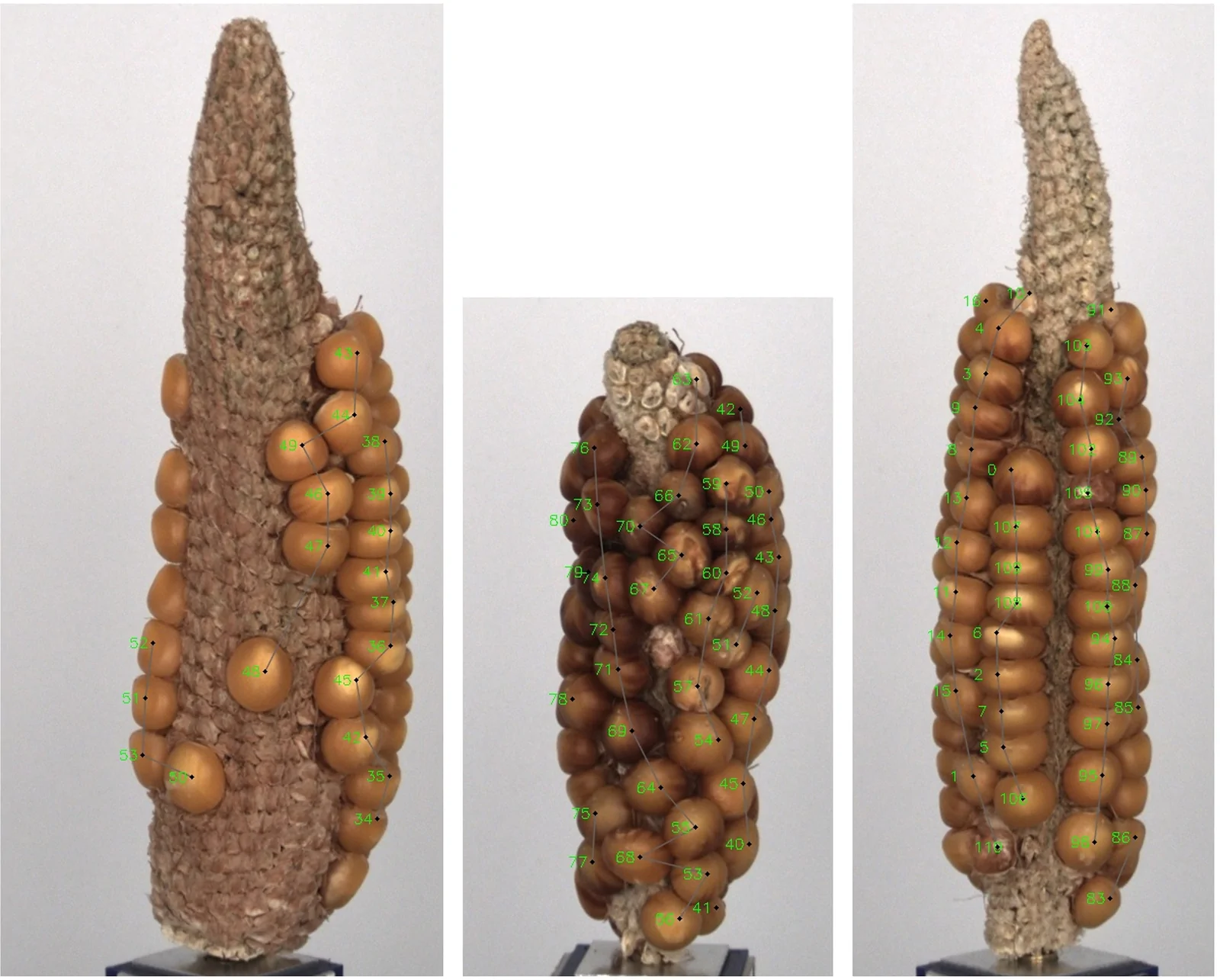
Challenging ears to predict kernel row number (KRN). Kernel positions are displayed as green ID numbers and black points on the undistorted RGB images. Grey lines indicate detected kernel rows. Only detections that are closer to the camera than the central ear axis at given *z*-coordinate are displayed.

Despite the moderate R^2^ of 0.82 and 0.77, very low MAPE of 3.1% and 2.5% could be achieved for EW and KRN, respectively, which indicates rather low variability of these traits in the dataset. This was especially expected for KRN as this trait can only be expressed as even numbers and does not vary much in maize genotypes. However, three serious outliers could be observed where at maximum four kernel rows were predicted by the ear scanning pipeline (Fig. 4). As shown in Fig. 7, the ears with abnormal morphology either exhibit missing kernel rows (left and right panels) or highly irregular kernel arrangements (centre panel). Both characteristics appear to limit the performance of the proposed clustering-based approach for predicting KRN. These challenges may be explained by the DBSCAN clustering being restricted to a central slab along the ear's z-axis. While human observers can identify the ear region in which the greatest number of kernel rows is visible, the proposed algorithm evaluates only the central region. Additionally, the ear scanning pipelines appears to be challenged in correctly assigning kernels to kernel rows of especially the ear in the centre image displayed in Fig. 7. The irregular position of the kernels leads to zig-zag patterns in the detected kernel row trajectories.

Previous studies have investigated ear phenotyping using 2D RGB image data [[7], [8], [9], [10], [11], [12],^14^,^15^], showing good accuracies for several traits that could be derived from a limited number of views like EL, EW and KN. However, with these approaches there has been no guarantee that all sides of the ears were included in the analysis. Nevertheless, Oury et al. [15] achieved very high accuracy for counting KN, by rotating the ear on motor-driven rolls, which provided a sufficient number of representative views. Yet, the detected traits were not oriented in the ears 3D space and 3D based traits such as EV, KRN and KRL were missing. EV has been predicted based on single 2D images by Ma et al. [14] achieving decent accuracy, yet not the complete ear was represented by this method. Similarly, Shi et al. [8] accurately counted KRN on single images where the ground truth was not based on the entire ear but was limited to the kernel rows that were visible on the image. In the study of Warman et al. [16] the ear was fixed on both the tip and the base to be rotated on a stepper motor while a video stream was captured by a camera. Due to accurate stepper motor movement, the authors created a panorama image of the entire ear without duplicated image parts after one revolution. This allowed accurate predictions of KN but no results on KRN and EV have been reported.

To our knowledge, Sun et al. [19] proposed the first approach to measure KRN, and KRL over the entire ear, rather than inferring these traits from a single or a limited number of views. This was achieved by scanning the ears with a structured-light scanner which enabled computing 3D point clouds of each ear. While this method proved accurate, the authors stated that scanning of the ear and reconstructing of the point cloud model took 5 to 7 min. In contrast, the ear-scanning pipeline proposed here only requires 30 s for capture of ear images and 47 s for computation of all traits. Possibly, the mounting of both the ear tip and ear base, as described in Sun et al. [19] is more time-consuming than mounting the ear only on a spike in the ear base, as described in this study. Here, the ear scanning pipeline allows to insert a spike adapter into the next ear while the scanning of another ear is still running. Once a scan is complete, the ear can be rapidly replaced by a new one. Additionally, the matching of 2D segmentation masks with the computed 3D voxel grid allows to measure KS, since the distance between camera and each detected kernel is known. However, a direct evaluation of KS compared to a manually measured trait remains to be done. KS could e.g. be compared to thousand seed weight (TSW), representing an important trait in maize breeding.

Altogether, previously proposed ear phenotyping pipelines either lacked to simultaneously measure multiple relevant ear traits (especially KRN and EV are missing in most pipelines), lacked to capture spatially oriented views distributed around the entire ear or were substantially slower than our approach. The here presented ear scanning pipeline addresses these limitations, providing a precise and rapid tool for multi-trait maize ear phenotyping.

### 4.4 Application of the ear scanning pipeline on ears from the P fertilization trial

The applicability of the here proposed ear scanning pipeline has been demonstrated by identifying coherent responses of the maize ear morphology to P deficiency. As can be seen in Fig. 6, there is no response to P-supply for traits that are related to kernel primordia initiation such as KN, KRN or KRL. Recent research has shown high broad-sense heritability for KRN [51] and KRL [52], highlighting the high genetic influence on these traits. Yet, KRN was shown to clearly respond to severe P-deficiency [53], possibly indicating that the environmental conditions from the here described P fertilization trial did not represent severe P-deficiency. Actually, no significant differences in biomass yield could be observed between P-fertilization levels in the field trial [21]. Herrmann et al. [21] suggested that challenging weather conditions in 2023 could have masked the effect of the P-fertilization. Nevertheless, a clear P-response was detected by the ear scanning pipeline for EW, EL, EV, KS and its related traits BW and BH. Noteworthy, the ratio of BW to BH was not influenced by P-supply, indicating no change in kernel shape.

Thus, for the genotype LG30258 under the given environmental conditions, it can be concluded that P does not influence the kernel positioning, kernel arrangement or kernel shape, while yield relevant traits such as EW, EL, EV, and KS are clearly increased due to P fertilization. While most publications on digital ear phenotyping rather focus on method development, this study is, to our knowledge, the first one applying a digital ear phenotyping pipeline in the field of plant nutrition. Nevertheless, it should be noted that these results originate from only 89 ears of one genotype grown at one location in one year. Whether the here reported phenotypic response to P-deficiency can be observed in other varieties, locations and years remains an open research question.

## 5 Conclusions

In this study, different strategies based on the machine vision models YOLO and SAM for instance segmentations of maize kernels have been evaluated. By combining YOLO8m for object detection and SAM for segmentation, a mAP_50-95_ of 0.720 and a DS of 0.940 were achieved. This strategy only requires fine-tuning YOLO8m with annotated bounding boxes making it resource efficient during development. During model inference, however, a single instance segmentation model may be more computationally efficient than combining YOLO and SAM.

Moreover, a novel approach of application-specific 2D-to-3D correspondence was proposed which exploits the geometry and spatial arrangement of maize kernels, allowing for efficiently matching single voxels to 2D kernel masks. This enabled tracking of kernel instances, scaling of ear traits measured on 2D images and measurement of EV. Consequently, the digital measurement of multiple traits describing ear morphology (EW, EL, EV) and traits describing kernel morphology (KN, KRN, KRL) has been established, with none of the traits differing from manual measurements by more than a MAPE of 5.7%. While KS was not compared to manual measurement in this study, the related thousand kernel weight would be a highly relevant trait to be implemented in future research.

In a P-fertilization trial the here proposed ear scanning pipeline enabled detecting significant P-responses on traits mainly affected by later ear development while traits related to kernel primordia initiation did not express significant responses. This highlights the need for accurate phenotyping pipelines capturing multiple traits simultaneously to unravel even intricate phenotypic responses to environmental conditions.

Overall, the here proposed ear scanning pipeline represents a novel and accurate high-throughput phenotyping solution with the potential to boost maize ear-related research in plant nutrition, plant breeding and further fields of plant science.

## CRediT authorship contribution statement

Leon H. Oehme: Conceptualization, Data curation, Formal analysis, Investigation, Methodology, Software, Validation, Visualization, Writing - original draft, Writing - review & editing. Tobias Schrag: Data curation, Investigation, Writing - review & editing. Peng Qi: Conceptualization, Visualization, Writing - review & editing. Bastian Stürmer-Stephan: Conceptualization, Methodology, Writing - review & editing. Khandoker T. Ahammad: Formal analysis, Writing - original draft, Writing - review & editing. Daniel Wanke: Investigation, Writing - review & editing. Tobias Würschum: Conceptualization, Funding acquisition, Project administration, Resources, Supervision, Writing - review & editing. Xiongkui He: Conceptualization, Funding acquisition, Project administration, Resources, Supervision, Writing - review & editing. Joachim Müller: Conceptualization, Funding acquisition, Project administration, Resources, Supervision, Writing - review & editing.

## Data availability

All relevant result data and source code will be made publicly available in the following repository upon publication: https://github.com/DerOehmer/maize-ear-voxel-phenotyping.

## Declaration of generative AI and AI-assisted technologies in the writing process

During the preparation of this work the authors used ChatGPT 5.3 (Open AI, Inc., San Francisco, U.S.) in order to improve written language. After using this tool/service, the authors reviewed and edited the content as needed and take full responsibility for the content of the published article.

## Funding

Funded by the Deutsche Forschungsgemeinschaft (DFG, German Research Foundation) – 328017493/GRK 2366 (Sino-German International Research Training Group AMAIZE-P).

## Declaration of competing interest

The authors declare the following financial interests/personal relationships which may be considered as potential competing interests:Leon H. Oehme reports financial support was provided by German Research Foundation. Peng Qi reports financial support was provided by German Research Foundation. Khandoker T. Ahammad reports financial support was provided by German Research Foundation. Daniel Wanke reports financial support was provided by German Research Foundation. Tobias Wuerschum reports financial support was provided by German Research Foundation. Xiongkui He reports financial support was provided by German Research Foundation. Joachim Mueller reports financial support was provided by German Research Foundation. If there are other authors, they declare that they have no known competing financial interests or personal relationships that could have appeared to influence the work reported in this paper.

## Figures and Tables

**Fig. A. 1:**
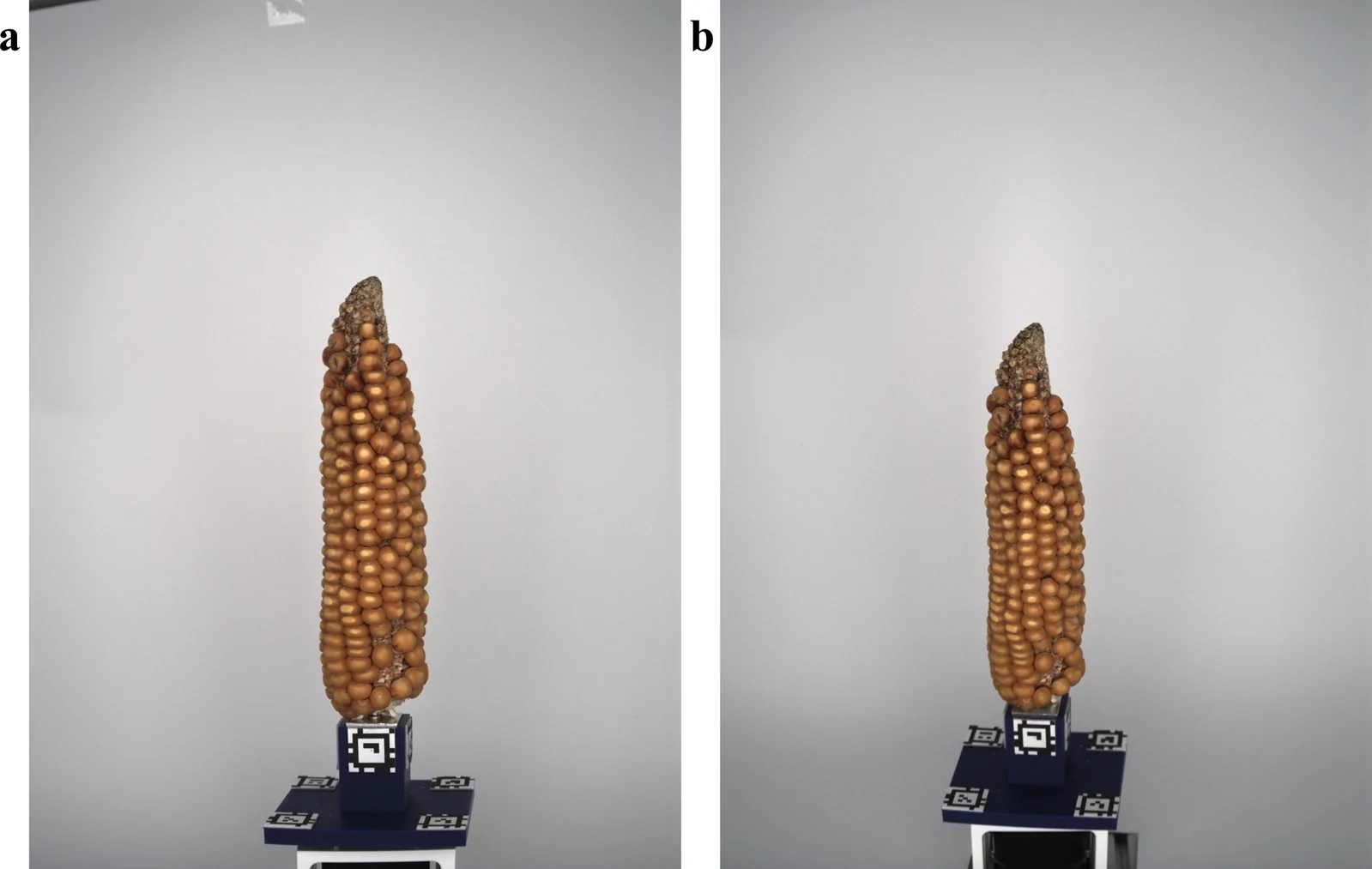
Raw images taken by the ear scanner. (a) Shows the lower camera view which is oriented horizontally while (b) shows the upper camera view being tilted downwards by *θ* = 17°.

**Fig. A. 2:**
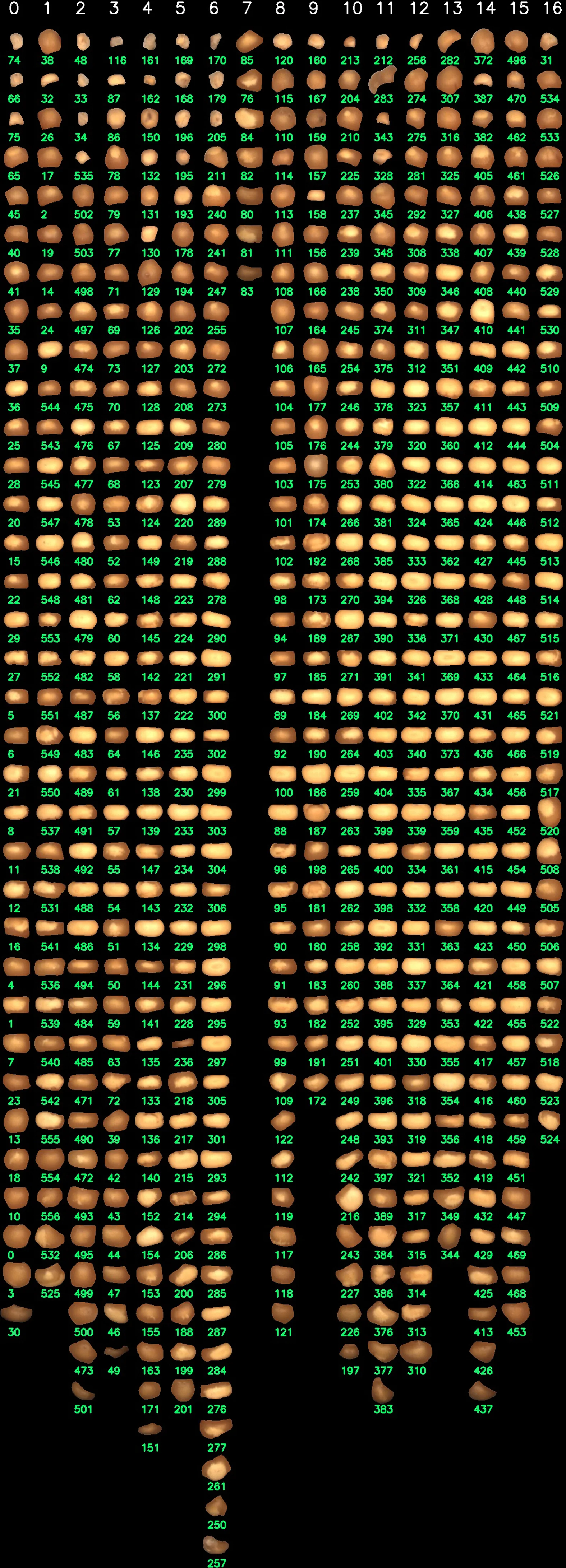
All detected maize kernels of the ear from Fig. 5 as identified by the ear scanner pipeline. The white numbers on top indicate the assigned kernel row. Green numbers below the kernels are unique identifiers. Kernels are shown as segments of the original RGB images, where they were first detected by YOLOv8m and SAM.
